# Oxygen-Loaded Nanodroplets Effectively Abrogate Hypoxia Dysregulating Effects on Secretion of MMP-9 and TIMP-1 by Human Monocytes

**DOI:** 10.1155/2015/964838

**Published:** 2015-03-23

**Authors:** Giulia Rossana Gulino, Chiara Magnetto, Amina Khadjavi, Alice Panariti, Ilaria Rivolta, Marco Soster, Monica Argenziano, Roberta Cavalli, Giuliana Giribaldi, Caterina Guiot, Mauro Prato

**Affiliations:** ^1^Dipartimento di Oncologia, Università di Torino, 10126 Torino, Italy; ^2^Istituto Nazionale di Ricerca Metrologica (INRIM), 10135 Torino, Italy; ^3^Dipartimento di Neuroscienze, Università di Torino, 10125 Torino, Italy; ^4^Dipartimento di Scienze della Salute, Università di Milano Bicocca, 20900 Monza, Italy; ^5^Dipartimento di Scienza e Tecnologia del Farmaco, Università di Torino, 10125 Torino, Italy; ^6^Dipartimento di Scienze della Sanità Pubblica e Pediatriche, Università di Torino, 10126 Torino, Italy

## Abstract

Monocytes play a key role in the inflammatory stage of the healing process. To allow monocyte migration to injured tissues, the balances between secreted matrix metalloproteinases (MMPs) and their inhibitors (TIMPs) must be finely modulated. However, a reduction of blood supply and local oxygen tension can modify the phenotype of immune cells. Intriguingly, hypoxia might be targeted by new effective oxygenating devices such as 2H,3H-decafluoropentane- (DFP-) based oxygen-loaded nanodroplets (OLNs). Here, hypoxia effects on gelatinase/TIMP release from human peripheral monocytes were investigated, and the therapeutic potential of dextran-shelled OLNs was evaluated. Normoxic monocytes constitutively released ~500 ng/mL MMP-9, ~1.3 ng/mL TIMP-1, and ~0.6 ng/mL TIMP-2 proteins. MMP-2 was not detected. After 24 hours, hypoxia significantly altered MMP-9/TIMP-1 balance by reducing MMP-9 and increasing TIMP-1, without affecting TIMP-2 secretion. Interestingly OLNs, not displaying toxicity to human monocytes after cell internalization, effectively counteracted hypoxia, restoring a normoxia-like MMP-9/TIMP-1 ratio. The action of OLNs was specifically dependent on time-sustained oxygen diffusion up to 24 h from their DFP-based core. Therefore, OLNs appear as innovative, nonconventional, cost-effective, and nontoxic therapeutic tools, to be potentially employed to restore the physiological invasive phenotype of immune cells in hypoxia-associated inflammation.

## 1. Introduction

The innate immune system provides the first line of defense against exogenous or endogenous danger signals, promoting a protective inflammatory response that evolves through different phases, from initiation and full inflammation to resolution and reestablishment of tissue integrity. In this perspective, inflammation has been described as an adaptive response to tissue malfunction or homeostatic imbalance [1]. However, the inflammatory activities are potentially harmful to the host; therefore, they need to be tightly controlled to prevent excessive tissue damage [2]. Human monocytes, constituting almost 10% of the total leukocytes, play a central role in the different stages of the inflammatory response, including antigen recognition and presentation, initiation of the adaptive immune response, and regulation of healing processes [3]. This wide spectrum of activities requires monocyte migration to the injured tissues and their fast adaptation to the changing microenvironment.

To allow extravasation from the blood vessels and migrate into the tissues, monocytes secrete several classes of proteins, including matrix metalloproteinases (MMPs). MMPs, a family of proteolytic enzymes secreted as latent zymogens activated locally by other proteases and inhibited by their secreted endogenous inhibitors (tissue inhibitor of metalloproteinases (TIMPs)) can collectively process all the components of the basement membrane and the extracellular matrix [4]. In addition, MMPs can cleave other molecules, including cytokines, chemokines, growth factors, enzymes, and membrane-bound proteins, thus promoting their activation, inhibition, degradation, or shedding and playing pivotal roles during inflammation [5, 6].

On the other hand, hypoxia, which is functionally defined as the inability of oxygen delivery to meet oxygen demands of the tissue [7], has been demonstrated in a variety of acute and chronic inflammatory sites, including chronic wounds, sites of bacterial infection, myocardial infarcts, the synovium in rheumatoid arthritis patients, and the arterial intima in atherosclerotic lesions [8]. Depending on its duration and severity, hypoxia can drive inflammation and aggravate cellular and tissue injury by inducing monocyte recruitment and causing their accumulation in hypoxic regions [9]. Hypoxia profoundly affects monocyte morphology, viability, and functionality; even more so, it alters the expression of surface molecules and release of soluble molecules, including cytokines, MMPs, and TIMPs [10].

To counteract tissue hypoxia, in recent years intensive research has been carried on to develop new oxygen carriers, either based on hemoglobin, developed as cell-free suspensions [11, 12] or on perfluorocarbons, carrying molecular oxygen without actually binding it, thus favoring gas exchange [13]. Among the alternative options currently under investigation, perfluoropentane-based oxygen-loaded nanobubbles [14, 15] and 2H,3H-decafluoropentane- (DFP-) based nanodroplets [16, 17], both coated with biocompatible polysaccharides such as chitosan or dextran, have been recently reported to deliver both* in vitro* and* in vivo* clinically relevant oxygen amounts, thus acting as efficient, biocompatible, and stable oxygen delivery systems. In particular, the nanometer size displays several advantages on a therapeutic level: (i) in accordance with Laplace's law, the smaller the bubble radius, the higher the oxygen partial pressure; (ii) the carriers are likely to pass through the nanosized interendothelial gaps of fenestrated capillaries; (iii) when needed, oxygen release can be easily promoted upon complementary ultrasound administration [17].

In the present work, new dextran-shelled oxygen-loaded nanodroplets (OLNs), which have recently been developed, characterized, and patented by our group [17], were challenged for their ability to counteract hypoxia in human monocytes isolated from peripheral blood, in order to assess their potential suitability as therapeutics during inflammation. OLN cytotoxicity and cell viability, as well as cellular uptake, were evaluated. Then, hypoxia and OLN effects on the secretion of gelatinases (MMP-9 and MMP-2) and their inhibitors (TIMP-1 and TIMP-2) by human monocytes were analyzed.

## 2. Materials and Methods

### 2.1. Materials

All materials were from Sigma-Aldrich (St. Louis, MO), aside from those listed below. Sterile plastics were from Costar (Cambridge, UK); Panserin 601 monocyte medium was from PAN Biotech (Aidenbach, Germany); cell culture medium RPMI 1640, TRIzol, M-MLV, oligo-dT, sense and anti-sense primers and Platinum Taq DNA Polymerase were from Invitrogen (Carlsbad, CA); DNA-free kit was from Ambion (Austin, TX); Beacon Designer 2.1 software was from Premier Biosoft International (Palo Alto, CA); dNTPs were from Applied Biosystem (Foster City, CA); ELISA kits for hMMP-9 (CAT# ELH-MMP9-001), hTIMP-1 (CAT# ELH-TIMP1-001), and hTIMP-2 (CAT# ELH-TIMP2-001) were from RayBiotech (Norcross, GA); iCycler iQ Real Time Detection System Software version 3.0, electrophoresis reagents, and computerized densitometer Geldoc were from Bio-rad Laboratories (Hercules, CA); Synergy HT microplate reader was from Bio-Tek Instruments (Winooski, VT); recombinant proMMP-9 and MMP-9 were kindly gifted by Professor Ghislain Opdenakker and Professor Philippe Van den Steen (Catholic University of Leuven, Belgium); ethanol (96%) was obtained from Carlo Erba (Milan, Italy); Epikuron 200 (soya phosphatidylcholine 95%) was from Degussa (Hamburg, Germany); palmitic acid, decafluoropentane (DFP), dextran sodium salt (100 kDa), and polyvinylpyrrolidone (PVP) were from Fluka (Buchs, Switzerland); ultrapure water was obtained using a 1-800 Millipore system (Molsheim, France); Ultra-Turrax SG215 homogenizer was from IKA (Staufen, Germany); Stratalinker UV Crosslinker 1800 was from Stratagene (La Jolla, CA); Delsa Nano C analyzer was from Beckman Coulter (Brea, CA); Miniscope 100 EPR spectrometer was from Magnettech (Berlin, Germany); Philips CM10 instrument was from Philips (Eindhoven, Netherlands); Formvar-coated copper grid was from Polysciences Europe GmbH (Eppelheim, Germany); XDS-3FL microscope was from Optika (Ponteranica, Italy); polarizing microscope was from Spencer Lens Company (Buffalo, NY); Discovery HR1 Hybrid Rheometer was from TA instruments (Milan, Italy).

### 2.2. Preparation of Dextran-Shelled OLN and Control Solutions

OLNs, oxygen-free nanodroplets (OFNs), and oxygen-saturated solution (OSS) were prepared as previously described [17]. Briefly, 1.5 mL DFP, 0.5 mL polyvinylpyrrolidone, and 1.8 mL Epikuron 200 (solved in 1% w/v ethanol and 0.3% w/v palmitic acid solution) were homogenized in 30 mL phosphate-buffered saline (PBS) solution (pH 7.4) for 2 min at 24000 rpm by using Ultra-Turrax SG215 homogenizer. For OLNs, the solution was saturated with O_2_ for 2 min. Finally, 1.5 mL dextran or rhodamine B-labeled dextran solution was added drop-wise whilst the mixture was homogenized at 13000 rpm for 2 min. For OFN and OSS PBS formulations, OLN preparation protocol was applied omitting O_2_ or dextran/DFP addition, respectively. After manufacturing, OLNs, OFNs, and OSS were sterilized through UV-C exposure for 20 min. Thereafter, UV-C-treated materials were incubated with cell culture RPMI 1640 medium in a humidified CO_2_/air-incubator at 37°C up to 72 h, not displaying any signs of microbial contamination when checked by optical microscopy. Moreover, UV-C-sterilized O_2_-containing solutions underwent further analyses through O_3_ measurement and electron paramagnetic resonance (EPR) spectroscopy, showing no O_3_ generation and negligible singlet oxygen levels immediately after UV-C exposure.

### 2.3. Characterization of Dextran-Shelled OLN and Control Solutions

The morphology of nanodroplet formulations was determined by transmitting electron microscopy (TEM) and by optical microscopy. TEM analysis was carried out using a Philips CM10 instrument, whereas optical microscopy was carried out using a XDS-3FL microscope. For TEM analysis nanodroplet formulations were dropped onto a Formvar-coated copper grid and air-dried before observation. Furthermore, average diameters and shell thickness were elaborated from TEM images. Sizes, polydispersity indexes, and zeta potentials of nanodroplets were determined by dynamic light scattering using Delsa Nano C instrument, displaying a (0.6 nm–7 *μ*m) range for measurements of particle size distribution. The polydispersity index indicates the size distribution within a nanodroplet population. For zeta potential determination, formulation samples were placed into an electrophoretic cell, where an electric field of approximately 30 V/cm was applied. The electrophoretic mobility was converted into zeta potential using the Smoluchowski equation [18]. The refractive indexes of OLN and OFN formulations were calculated through a polarizing microscope. The viscosity and the shell shear modulus were determined through a Discovery HR1 Hybrid Rheometer. Immediately after preparation, oxygen content of OLNs and OSS was evaluated for characterization purposes by adding known amounts of sodium sulfite and measuring generated sodium sulfate, according to the reaction:

(1) $$\begin{array}{c} 2{\text{Na}}_{2}{\text{SO}}_{3}+{\text{O}}_{2}\to 2{\text{Na}}_{2}{\text{SO}}_{4} \end{array}$$

The stability of formulations stored at 4°C, 25°C, or 37°C was evaluated over time up to 6 months by determining morphology, sizes, and zeta potential of nanodroplets by optical microscopy and light scattering.

### 2.4. Preparation and Handling of Monocytes

Human monocytes were separated by Ficoll centrifugation [19] from freshly collected buffy coats discarded from blood donations by healthy adult donors of both sexes provided by the local blood bank (AVIS, Associazione Volontari Italiani Sangue, Torino, Italy). Separated lymphocytes and monocytes were resuspended in RPMI medium and plated on six-well plates. Each well received 8 × 10^6^ cells. The plates were incubated in a humidified CO_2_/air-incubator at 37°C for 60 min. Thereafter, nonadherent cells were removed by three washes with RPMI and remaining adherent cells (~1 × 10^6^ monocytes/well) were incubated overnight at 37°C. The day after, wells were washed with RPMI and Panserin 601 monocyte medium was added (2 mL/well). Before starting experiments, a preselection of cell populations was taken as a precautionary measure to reduce variability among donors, as previously described [20]. Briefly, cell cultures isolated through Ficoll separation were analyzed by flow cytometry. Only cell populations showing at least 70% monocytes were used for following experiments. Additionally, in order to avoid the use of NF-*κ*B preactivated monocytes, cells were analyzed by Real Time RT-PCR. in each cell preparation a cell aliquot was stimulated or not with LPS (1 *μ*g/mL) for 4 h, and TNF-*α* RNA production measured in lysates by Real Time RT-PCR. Total cellular RNA was isolated from monocytes by TRIzol, according to the manufacturer's instructions, and eluted in 20 *μ*L of diethyl pyrocarbonate water. To remove any contaminating DNA, RNA was treated with Ambion's DNA-free kit. Subsequently, 6 *μ*g of RNA was reverse transcribed into single-stranded cDNA using Moloney murine leukemia virus reverse transcriptase (200 U/*μ*L final concentration) and oligo(dT) (25 *μ*g/*μ*L final concentration). Real Time RT-PCR analysis was performed with iCycler iQ real-time RT-PCR Detection System apparatus and Chemidoc software, version 3.0 (Bio-Rad). A housekeeping gene glyceraldehyde-3-phosphate dehydrogenase (*GAPDH*) was used. Primer sequences were from the Bio-Rad library: forward, 5′-GAA GGT GAA GGT CGG AGT-3′ and reverse, 5′-CAT GGG TGG AAT CAT ATT GGA A-3′. For each 25-*μ*L PCR mix: 1 *μ*L of cDNA (corresponding to 10^5^ cells); 1.0 *μ*L of sense primer (10 *μ*M); 1.0 *μ*L of antisense primer (10 *μ*M); 0.5 *μ*L of dNTP (10 mM); 1.5 *μ*L of MgCl_2_ (50 mM); 1.25 U of Platinum* Taq*DNA Polymerase; 2.5 *μ*L of buffer (10X); 1.7 *μ*L of SYBR Green (stock 1 : 10,000); and 14.55 *μ*L of PCR-grade water were mixed together. DNA polymerase was preactivated for 2 min at 94°C, and the amplification was performed by 50 cycles (*TNF*) or 35 cycles (*GAPDH*) with denaturation at 94°C for 30 s, annealing at 60°C for 30 s and extension at 72°C for 30 s. Relative quantitation for* TNF*, expressed as fold variation over untreated control cells, was calculated after determination of the difference between CT of the given gene A (*TNF*) and that of the calibrator gene B (*GAPDH*) using the 2^−ΔΔCT^ method. Only unstimulated monocyte populations (NF-*κ*B-nonactivated cells) showing at least a gap of three PCR cycles of cDNA amplification between controls and LPS-stimulated cells were used for the subsequent experiments.

### 2.5. Evaluation of OLN Uptake by Human Monocytes

Human monocytes were plated in 24-well plates on glass coverslips and incubated in Panserin 601 medium for 24 h with/without 200 *μ*L rhodamine B-labeled OLNs in a humidified CO_2_/air-incubator at 37°C. After 4′,6-diamidino-2-phenylindole (DAPI) staining to visualize cells nuclei, fluorescence images were acquired by a LSM710 inverted confocal laser scanning microscope equipped with a Plan-Neofluar 63 × 1.4 oil objective that allowed a field view of at least 5 cells. Wavelength of 544 nm was used to detect OLNs and of 460 nm to detect the labeled nuclei. The acquisition time was 400 ms.

### 2.6. OLN Cytotoxicity Studies

The potential cytotoxic effects of OLN and control formulations were measured as the release of lactate dehydrogenase (LDH) from human adherent monocytes into the extracellular medium as previously described [21]. Briefly, cells were incubated in Panserin 601 medium for 24 h in the presence or absence of increasing doses (100–400 *µ*L) of OLNs or 200 *µ*L of OFNs or OSS, either in normoxic (20% O_2_) or in hypoxic (1% O_2_) conditions, in a humidified CO_2_/air-incubator at 37°C. Then, 1 mL of cell supernatants was collected and centrifuged at 13000 g for 2 min. Cells were washed with fresh medium, detached with trypsin/EDTA (0.05/0.02% v/v), washed with PBS, resuspended in 1 mL of TRAP (82.3 mM triethanolamine, pH 7.6), and sonicated on ice with a 10 s burst. 5 *µ*L of cell lysates and 50 *µ*L of cell supernatants were diluted with TRAP and supplemented with 0.5 mM sodium pyruvate and 0.25 mM NADH (300 *μ*L as a final volume) to start the reaction. The reaction was followed measuring the absorbance at 340 nm (37°C) with Synergy HT microplate reader. Both intracellular and extracellular enzyme activities were expressed as *μ*mol of oxidized NADH/min/well. Finally, cytotoxicity was calculated as the net ratio between extracellular and total (intracellular + extracellular) LDH activities.

### 2.7. Cell Viability Studies

Cell viability was evaluated using 3-(4,5-dimethylthiazol-2-yl)-2,5-diphenyltetrazolium bromide (MTT) assay. Human adherent monocytes were incubated in Panserin 601 medium for 24 h with/without increasing doses (100–400 *µ*L) of OLNs or 200 *µ*L of OFNs or OSS, either in normoxic (20% O_2_) or hypoxic (1% O_2_) conditions, in a humidified CO_2_/air-incubator at 37°C. Thereafter, 20 *μ*L of 5 mg/mL MTT in PBS were added to cells for 3 additional hours at 37°C. The plates were then centrifuged, the supernatants discarded and the dark blue formazan crystals dissolved using 100 *μ*L of lysis buffer containing 20% (w/v) sodium dodecyl sulfate (SDS), 40% N,N-dimethylformamide (pH 4.7 in 80% acetic acid). The plates were then read on Synergy HT microplate reader at a test wavelength of 550 nm and at a reference wavelength of 650 nm.

### 2.8. Measurement of the Levels of Latent and Active Forms of MMP-9 Protein in Cell Supernatants

The levels of latent and active forms of MMP-9 were evaluated by gelatin zymography in the cell supernatants as previously described [22]. Briefly, human adherent monocytes (1 × 10^6^ cells/well) were incubated in Panserin 601 medium for 24 h with/without 200 *µ*L of OLNs, OFNs, or OSS, either in normoxic (20% O_2_) or hypoxic (1% O_2_) conditions, in a humidified CO_2_/air-incubator at 37°C. Thereafter, 15 *μ*L cell supernatants/lane were loaded on 8% polyacrylamide gels containing 0.1% gelatin under nondenaturing and nonreducing conditions. Following electrophoresis, gels were washed at room temperature for 2 h in milliQ water containing 2.5% (v/v) Triton-X100 and incubated for 18 h at 37°C in a collagenase buffer containing (mM): NaCl, 200; Tris, 50; CaCl_2_, 10; and 0.018% (v/v) Brij 35, pH 7.5, with or without 5 mM EDTA to exclude nonspecific bands. At the end of the incubation, the gels were stained for 15 min with Coomassie blue (0.5% Coomassie blue in methanol/acetic acid/water at a ratio of 30 : 10 : 60). The gels were destained in milliQ water. Densitometric analysis of the bands, reflecting the total levels of latent and active forms of MMP-9, was performed using a computerized densitometer.

### 2.9. Measurement of MMP-9, TIMP-1, and TIMP-2 Production

Human adherent monocytes (1 × 10^6^ cells/well) were incubated in Panserin 601 medium for 24 h with/without 200 *µ*L of OLNs, OFNs, or OSS, either in normoxic (20% O_2_) or hypoxic (1% O_2_) conditions, in a humidified CO_2_/air-incubator at 37°C. Thereafter, cell supernatants were collected, and the levels of MMP-9, TIMP-1, and TIMP-2 were assayed in 100 *μ*L of monocyte supernatants by specific ELISA. Standard calibration curves were generated with rhMMP-9, rhTIMP-1, and rhTIMP-2, according to the manufacturer's instructions.

### 2.10. Statistical Analysis

For each set of experiments, data are shown as means + SEM (LDH, MTT, densitometry, and ELISA results) or as a representative image (confocal microscopy and gelatin zymography results) of three independent experiments with similar results. SEM was used since biological data were considered as inferential information (see [23] for an exhaustive review). All data were analyzed by a one-way analysis of variance (ANOVA) followed by Tukey's post hoc test (software: SPSS 16.0 for Windows, SPSS Inc., Chicago, IL).

## 3. Results

### 3.1. Characterization of OLND and Control Formulations

Before being employed in the subsequent experiments, all OLND preparations were characterized for morphology and shell thickness, by optical and transmitting electron microscopy (TEM); size, particle size distribution, polydispersity index and zeta potential, by dynamic light scattering; refractive index by polarizing microscopy; viscosity and shell shear modulus by rheometry; and oxygen content (before and after UV-C sterilization) through a chemical assay. Results were always in line with literature data [17]. Both OLNs and OFNs displayed spherical shapes. All sizes were in the nanometer range, with average diameters ranging from ~240 nm for OFNs to ~590 nm for OLNs. Zeta potentials were ~−25 mV. Refractive indexes were similar for both OLN and OFN formulations (~1.33). OLN formulation displayed a viscosity value of 1.59188  *e* − 3 Pa·s and a shear modulus value of 5.43  *e* − 2 mPa, calculated at a shear rate value of 150 s^−1^. OLNs displayed a good oxygen-storing capacity of about 0.40 mg/mL of oxygen before and after 20 min UV-C sterilization. Such oxygen amount was comparable with that of OSS.

### 3.2. OLN Effects on Human Monocyte Viability

**Figure 1:**
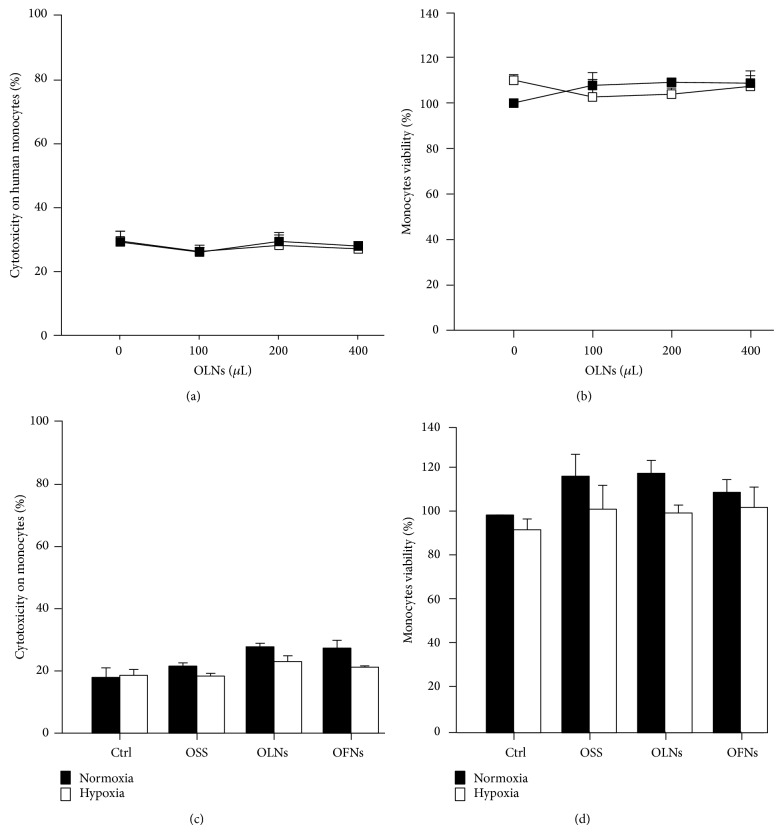
Hypoxia and OLN effects on human monocyte viability. Human adherent monocytes (10^6^ cells/2 mL Panserin 601 medium) were left untreated or treated with increasing doses (100–400 *μ*L) of OLNs or with 200 *μ*L OFNs or OSS for 24 h in normoxia (20% O_2_, black columns/squared-lines) or hypoxia (1% O_2_, white columns/squared-lines). After collection of cell supernatants and lysates, cytotoxicity percentage was measured through LDH assay (panels (a) and (c)), whereas cell viability percentage was measured through MTT assay (panels (b) and (d)). Results are shown as means + SEM from three independent experiments. Data were also evaluated for significance by ANOVA. No significant differences between normoxic and hypoxic control cells or between OLN-treated and untreated cells were observed (all panels).

OLN toxicity was evaluated by testing* in vitro* cultures of human adherent monocytes isolated from peripheral blood. As shown in Figure 1  (Figure 1(a): LDH assay; Figure 1(b): MTT assay), increasing volumes of OLN PBS suspensions (5, 10, and 20% v/v) were not toxic to monocytes (10^6^ cells per 2 mL Panserin 601 cell culture medium) both in normoxic (20% O_2_) and hypoxic (1% O_2_) conditions. Therefore, the intermediate OLN dosage (10% v/v) was chosen to perform the subsequent experiments. Toxicity of 10% v/v OFNs and OSS (controls) was also assayed (Figure 1(c): LDH assay; Figure 1(d): MTT assay), not showing any effect on viability of treated cells with respect to untreated cells. As expected, 0.5% Triton X-100, an effective cytotoxic agent employed as positive control, induced 100% cell death (not shown).

### 3.3. OLN Internalization by Human Monocytes

**Figure 2:**
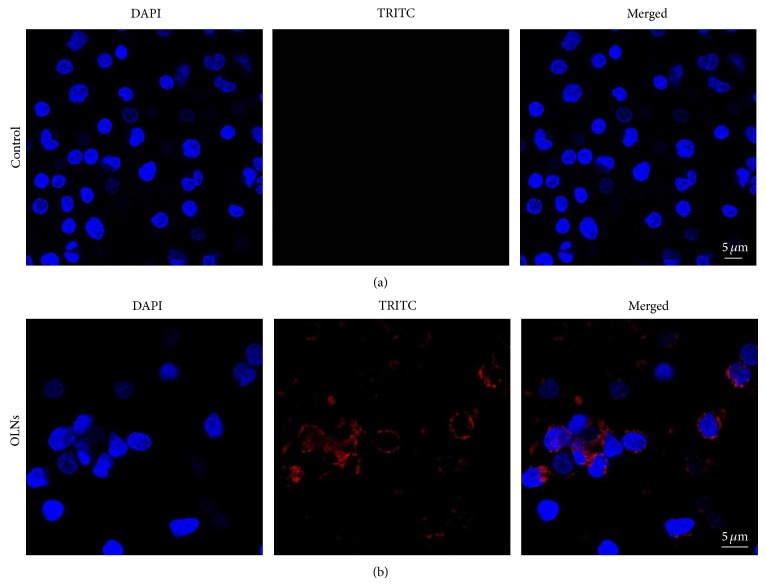
OLN internalization by human monocytes. Human adherent monocytes (10^6^ cells/2 mL Panserin 601 medium) were left untreated (a) or treated with 200 *μ*L rhodamine B-labeled dextran-shelled OLNs (b) for 24 h in normoxia (20% O_2_). After DAPI staining, cells were checked by confocal microscopy. Results are shown as representative images from three independent experiments. Left panels: cell nuclei after DAPI staining (blue). Central panels: rhodamine B-labeled dextran-shelled OLNs (red). Right panels: merged images. Magnification: 63x.

To check whether OLNs were uptaken by human adherent monocytes isolated from peripheral blood, a confocal microscopy approach was chosen. Human adherent monocytes (10^6^ cells per 2 mL Panderin 601 cell culture medium) were incubated with 10% v/v rhodamine B-labeled dextran-shelled OLN PBS suspensions for 24 h in normoxic conditions. As shown in Figure 2, OLNs were internalized by human monocytes.

### 3.4. Hypoxia and OLN Effects on MMP-9 Secretion by Human Monocytes

**Figure 3:**
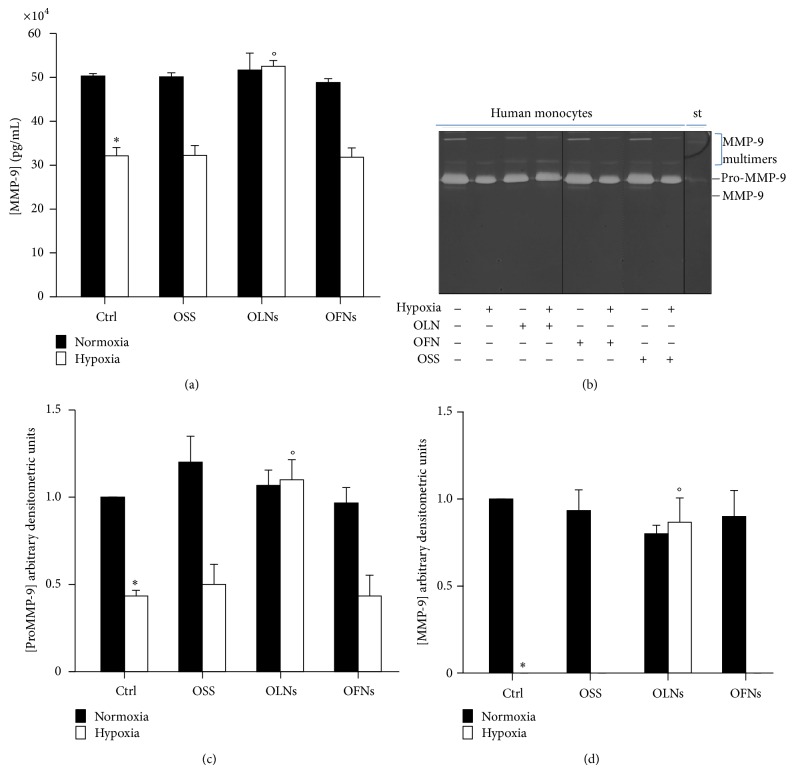
Hypoxia and OLN effects on MMP-9 secretion by human monocytes. Human adherent monocytes (10^6^ cells/2 mL Panserin 601 medium) were left untreated or treated with 200 *μ*L OLNs, OFNs, or OSS for 24 h in normoxia (20% O_2_; panels (a), (c), and (d): black columns; panel (b): odd lanes) or hypoxia (1% O_2_; panels (a), (c), and (d): white columns; panel (b): even lanes). After collection of cell supernatants, MMP-9 protein levels were quantified by ELISA (panel (a)), whereas MMP-9 latent/active forms were analyzed by gelatin zymography (panel (b)) and subsequent densitometry (panels (c)-(d)). For gelatin zymography, recombinant human MMP-9 (83 kDa) was employed as a standard marker (st). Results are shown as means + SEM (panels (a), (c)-(d)) or as a representative gel (panel (b)) from three independent experiments. ELISA and densitometric data were also evaluated for significance by ANOVA: ∗ versus normoxic control cells: *P* < 0.0001 (panel (a)), *P* < 0.02 (panel (c)), and *P* < 0.0001 (panel (d)); ° versus hypoxic control cells: *P* < 0.0001 (panel (b)), *P* < 0.005 (panel (c)), and *P* < 0.0001 (panel (d)).

After incubating for 24 h human adherent monocytes isolated from peripheral blood (10^6^ cells per 2 mL cell culture medium) with or without 10% v/v OLNs, OFN, or OSS, both in normoxic (20% O_2_) and hypoxic (1% O_2_) conditions, MMP-9 secretion into cell supernatants was evaluated. Results are shown in Figure 3. ELISA analysis (Figure 3(a)) displayed that normoxic untreated monocytes constitutively secreted ~500000 pg/mL MMP-9. Complementary investigation by gelatin zymography (Figure 3(b)) and subsequent densitometry (Figures 3(c) and 3(d)) revealed that both latent and activated forms of MMP-9 were released by normoxic monocytes. Furthermore, hypoxia was shown through both analyses to significantly alter MMP-9 secretion, lowering the 92 kDa proMMP-9 protein levels and fully inhibiting the release of the 83 kDa activated form. OLNs—but not OFNs and OSS—fully reversed hypoxia effects, restoring normoxic proMMP-9 and MMP-9 secretion. Notably, neither gelatin zymography nor ELISA analyses detected any MMP-2 protein amounts in monocyte supernatants.

### 3.5. Hypoxia and OLN Effects on TIMP Secretion by Human Monocytes and MMP-9/TIMP-1 Balances

**Figure 4:**
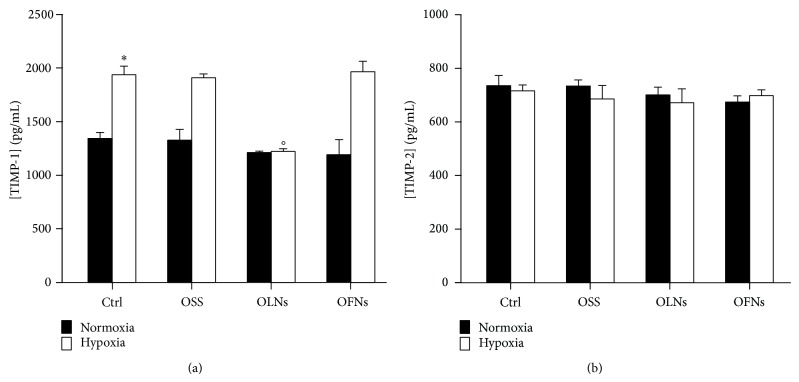
Hypoxia and OLN effects on protein levels of gelatinase inhibitors (TIMP-1 and TIMP-2) secreted by human monocytes. Human adherent monocytes (10^6^ cells/2 mL Panserin 601 medium) were left untreated or treated with 200 *μ*L OLNs, OFNs, or OSS for 24 h in normoxia (20% O_2_; black columns, both panels) or hypoxia (1% O_2_; white columns, both panels). After collection of cell supernatants, TIMP-1 (panel (a)) and TIMP-2 (panel (b)), protein levels were quantified by ELISA. Results are shown as means + SEM from three independent experiments. Data were also evaluated for significance by ANOVA: ∗ versus normoxic control cells: *P* < 0.02 (panel (a)) and *P* not significant (panel (b)); ° versus hypoxic control cells: *P* < 0.02 (panel (a)) and *P* not significant (panel (b)).

**Figure 5:**
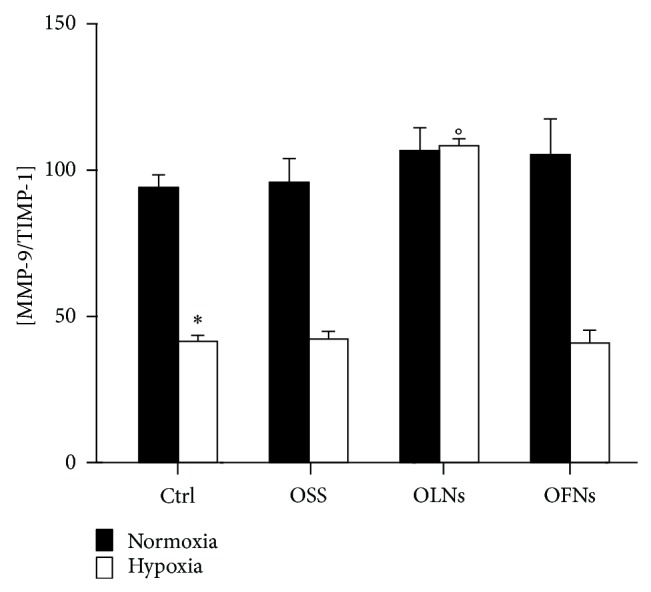
Hypoxia and OLN effects on MMP-9/TIMP-1 balances upon secretion by human monocytes. MMP-9/TIMP-1 stoichiometric ratio was calculated after results from ELISA investigation (see Figures 3 and 4). Results are shown as means + SEM from three independent experiments. Data were also evaluated for significance by ANOVA: ∗ versus normoxic control cells: *P* < 0.0001; ° versus hypoxic control cells: *P* < 0.0001.

Human adherent monocytes (10^6^ cells per 2 mL cell culture medium) were incubated for 24 h with or without 10% v/v OLNs, OFN, or OSS, both in normoxic (20% O_2_) and hypoxic (1% O_2_) conditions. Thereafter, the secretion of TIMP-1 and TIMP-2 was evaluated by ELISA. As shown in Figure 4, normoxic untreated monocytes constitutively released ~1300 pg/mL TIMP-1 and ~600 pg/mL TIMP-2. Hypoxia significantly increased by almost 45% the secreted levels of TIMP-1 while did not affect TIMP-2 production. OLNs—but not OFNs and OSS—effectively abrogated hypoxia effects, restoring physiological TIMP-1 amounts also in hypoxic culturing conditions. Consequently, the balance between MMP-9 and its inhibitor was calculated. As shown in Figure 5, hypoxia significantly affected MMP-9/TIMP-1 stoichiometric ratio, which was reduced by ~50% with respect to cells cultured in normoxic conditions. OLNs—but not OFNs and OSS—effectively counteracted hypoxia effects, restoring the physiological MMP-9/TIMP-1 ratio to a value similar to that observed in normoxia.

## 4. Discussion

The present study investigated the* in vitro* effects of hypoxia on the secretion of gelatinases and their inhibitors by human adherent monocytes isolated from peripheral blood and explored the therapeutic potential of innovative and nonconventional dextran-shelled and DFP-cored OLNs to counteract hypoxia-dependent dysregulation of the release of these molecules.

Human monocytes were cultured in normoxic conditions or exposed to controlled hypoxia. Normoxic monocytes constitutively released both latent and active forms of MMP-9 (but not MMP-2), as well as TIMP-1 and TIMP-2, in line with previous reports on human monocytes [24–27]. Hypoxia strongly impaired MMP-9/TIMP-1 balance by reducing MMP-9 secretion and increasing TIMP-1 release. Notably, hypoxic monocytes did not undergo apoptosis or necrosis during the observational period, in accordance with data from previous studies [28]. Therefore, the reduced proMMP-9 and MMP-9 levels in cell supernatants were not caused by a decreased number of cells but appeared as a direct consequence of monocyte adaptation to the hypoxic stress. Interestingly, it has been previously reported that in human monocytes prolonged hypoxia leads to the accumulation of intracellular proMMP-9 via cytoskeletal-mediated inhibition of its trafficking, thus reducing its secreted levels [28]. Lahat and colleagues have also reported that hypoxia reduces by three- to four-fold the TIMP-2 secretion from human primary monocytes as well as U937 and THP-1 monocyte cell lines, by inhibiting TIMP-2 transcription via mechanisms involving SP-1 transcription factor [29]. However, in our experiments, hypoxia did not affect TIMP-2 levels in monocyte supernatants. The discrepancy between the present results and those obtained by Lahat's group appears as a likely consequence of the different duration of monocyte incubation in hypoxic conditions in either study (24 h versus 72 h).

Once we determined the entity of hypoxia-dependent dysregulation of secreted MMP-9/TIMP-1 balances in human adherent monocytes, OLNs were challenged as potential tools to counteract hypoxia effects. These innovative and nonconventional gas nanocarriers, recently developed by our group [16, 17], display a core structure based on DFP, a liquid fluorocarbon which can carry molecular oxygen without actually binding it and favors gas exchange [30]. Fluorocarbons such as DFP are extremely stable, biologically inert, and rapidly excreted into the expired air in a nonmetabolized form after injection into the bloodstream, as emerged from the available studies on their toxicity, absorption, distribution, and excretion [31, 32]. On the other hand, dextran represents the main constituent of nanodroplet shells. Toxicological studies showed that dextran, along with the products of its mechanochemical processing, can be classified as class 4 (low-toxicity) substance [33]. Dextran-based hydrogels are currently used as matrices in tissue engineering, without showing signs of inflammation* in vivo* [11, 34, 35].

Before being employed in biological experiments, all dextran-shelled/DFP-cored nanodroplet preparations were meticulously characterized for morphology, average diameters, size distribution, polydispersity index, zeta potential, refractive index, viscosity, shell shear modulus, and oxygen content (both before and after UV-C sterilization). For all these parameters, results were consistent with those previously obtained and recently published [17]: nanodroplets displayed spherical shapes, nanometric sizes, and negative charges, as well as similar viscosity and shell shear modulus. Nanodroplet formulations also proved to be physically stable for the steric repulsion of the polymer chains, as assessed by repeated measurements of their sizes and zeta potential by dynamic light scattering over time up to 6 months. As expected, OLN solutions also displayed a good oxygen-storing capacity (0.4 mg O_2_/mL) either before or after UV-C sterilization, a procedure that was not accompanied by ozone and singlet oxygen generation.

In the present work, human monocytes were incubated in normoxic and hypoxic conditions with OLN and control (OFN and OSS) preparations for 24 h. OLNs were uptaken by human monocytes, not displaying any cytotoxic effects, either in normoxia or hypoxia. Intriguingly, OLNs fully abrogated hypoxia-dependent dysregulating effects on MMP-9 and TIMP-1 secretion. Even in hypoxic culturing conditions, OLN-treated monocytes secreted normoxia-like levels of MMP-9, TIMP-1, and TIMP-2, and the physiological balance between MMP-9 and TIMP-1 was restored.

Apparently, such an effect was specifically dependent on time-sustained oxygen release from the DFP-based inner core of OLNs, since it was not reproduced after treatment with OFNs and OSS. These observations are in line with previous data obtained from investigation on the dynamics of oxygen release from OLNs, OFNs, and OSS. Indeed, OLNs are reportedly able to release significant and clinically relevant amounts of oxygen into hypoxic environments in a time-sustained manner, opposite to OSS, which releases oxygen only transiently, and to OFNs, not releasing oxygen at all [16, 17]. In particular, 10% v/v OLNs, OFNs, and OSS have been comparatively challenged for their ability to release oxygen into Panserin 601 cell culturing medium and monitored by oxymetry for 24 h. Although both OLNs and OSS induced an immediate peak in oxygen release, only OLNs released high oxygen amounts in a time-sustained manner for all the observational 24 h-period, whereas OSS effect quickly vanished after a few hours and OFNs did not release significant amounts of oxygen [36]. These data appear encouraging, since they suggest that a topical administration of multiple doses (e.g., twice a day) of OLNs might be effective. Nevertheless, further preclinical and clinical studies on OLN potential as therapeutic tools to target specific ischemic areas are certainly needed. Intriguingly, OLNs can be further functionalized after conjugation with specific antibodies to the outer polysaccharidic shell [16, 17]. Furthermore, in a recent work, OLNs have also been shown to counteract* in vitro* the effects of hypoxia on the secretion of gelatinases and TIMPs by human keratinocytes [36]. Since both monocytes and keratinocytes play crucial roles throughout the overlapping phases of wound healing (hemostasis, inflammation, cellular proliferation, and remodeling) [37], this evidence supports the hypothesis that OLNs could be hopefully employed to promote tissue repair and regeneration in hypoxic chronic wounds.

## 5. Conclusions

The present work shows that the balance between MMP-9 and TIMP-1 in human adherent monocytes is dramatically altered by hypoxia. Intriguingly, dextran-shelled/DFP-cored OLNs—a new platform of oxygen nanocarriers recently developed by our group—are effective in counteracting the effects of hypoxia, restoring normoxia-like MMP-9 and TIMP-1 levels. These results, combined with the intrinsic benefits of nanodroplets (size, charge, stability, controlled release, and suitability for further drug functionalization, drug loading, or encapsulation), support the proposal that OLNs may serve as innovative, nonconventional, cost-effective, and nontoxic therapeutic tools to be potentially employed for restoring the physiological invasive phenotype of immune cells in hypoxia-associated inflammation. Based on the present* in vitro* evidence, future preclinical studies to translate this technology to clinical practice are encouraged.
